# Relationship between tau-PET and quantitative susceptibility mapping in atypical Alzheimer’s disease

**DOI:** 10.3389/fnagi.2025.1615718

**Published:** 2025-07-03

**Authors:** Neha Singh-Reilly, Ryota Satoh, Jonathan Graff-Radford, Mary M. Machulda, Val J. Lowe, Keith A. Josephs, Jennifer L. Whitwell

**Affiliations:** ^1^Department of Radiology, Mayo Clinic, Rochester, MN, United States; ^2^Department of Neurology, Mayo Clinic, Rochester, MN, United States; ^3^Department of Psychiatry and Psychology, Mayo Clinic, Rochester, MN, United States

**Keywords:** atypical Alzheimer’s disease, QSM, iron burden, tau-PET, tau accumulation

## Abstract

**Background:**

Iron is an important component in neurofibrillary tangles, is known to co-localize with tangles in Alzheimer’s disease (AD) and can be measured using quantitative susceptibility mapping (QSM). However, it is unclear if iron measured using QSM is regionally related to tau in atypical presentations of AD.

**Methods:**

Forty patients with atypical AD underwent a 3 T magnetic resonance imaging (MRI) scan with a five-echo gradient echo sequence to calculate QSM, Aβ, and [^18^F] AV-1451 positron emission tomography (PET). The relationship between QSM and tau-PET was assessed using voxel-based regression analysis using whole brain VoxelStats and region-of-interest (ROI)-based Spearman’s correlation analyses using cortical and subcortical ROIs.

**Results:**

At the voxel-level, positive correlations between tau-PET and QSM were only observed in the left caudate. At the ROI-level, a positive association was observed between tau-PET and susceptibility in the occipital lobe and a negative association was observed between substantia nigra susceptibility and occipital tau-PET uptake, although these findings did not survive correction for multiple comparisons.

**Discussion:**

Our data provides little evidence that regional tau-PET uptake is related to susceptibility changes, suggesting that iron deposition may not be directly associated with tau accumulation in atypical AD.

## 1 Introduction

Iron is known to bind to tau proteins (Sayre et al., 2000), induce tau protein phosphorylation, and play an important role in the accumulation of hyperphosphorylated tau to form neurofibrillary tangles (Yamamoto et al., 2002). In postmortem studies, iron deposition in the cortex, particularly the temporal cortex, has been associated with the severity of beta amyloid (Aβ) and tau pathology in Alzheimer’s disease (AD), supporting an important mechanistic role for iron in AD (van Duijn et al., 2017; Bulk et al., 2018; Ayton et al., 2021). Iron burden in the inferior temporal cortex, specifically, has been associated with the formation of neurofibrillary tangles (Ayton et al., 2021), with the temporal lobe showing severe iron accumulation and tau burden compared to other cortical regions (Bulk et al., 2018).

Quantitative susceptibility mapping (QSM) is a relatively new, non-invasive, magnetic resonance imaging (MRI) technique to capture cerebral iron. It can provide estimates of the local magnetic susceptibility in tissues at the voxel-level (Langkammer et al., 2012) and can detect paramagnetic (positive magnetic susceptibility) iron burden (Wang and Liu, 2015) and diamagnetic (negative magnetic susceptibility) myelin loss (Liu et al., 2015) in the brain. Several previous studies have assessed differences in susceptibility signatures in postmortem tissue and concluded that the bulk of the magnetic susceptibility in gray matter structures in AD is contributed by the iron content (Hallgren and Sourander, 1958; Langkammer et al., 2012; Tiepolt et al., 2020). There is evidence that QSM susceptibility in the temporal lobe is associated with tau deposition measured using positron emission tomography (PET) across the AD continuum (including cognitively unimpaired and impaired individuals) (Spotorno et al., 2020).

However, it is unclear whether iron deposition is related to the heterogeneity in tau deposition observed across atypical clinical presentations of AD. Atypical clinical presentations of AD are non-amnestic and typically characterized by the presence of visual, language, behavioral, executive, or motor difficulties (Galton et al., 2000; Graff-Radford et al., 2021). Spatial patterns of cortical tau accumulation on PET differ across the different variants of atypical AD, with prominent burden in the posterior regions in the visual variant, left dominant temporal regions in the language variant, temporo-parietal regions in the behavioral/executive variant and temporo-parieto-occipital regions with involvement of the sensorimotor regions in the motor variant (Ossenkoppele et al., 2016; Tetzloff et al., 2018). We have previously showed differing patterns of abnormal magnetic susceptibility in the cortex of the visual and language variants of atypical AD, specifically in the temporo-parieto-occipital regions and putamen in the visual variant and in the temporo-occipital regions, caudate, putamen and substantia nigra in the language variant (Singh et al., 2022). Bearing in mind, that iron is known to bind to tau, induce phosphorylation, support hyperphosphorylated tau accumulation and tangle formation in amnestic AD (Sayre et al., 2000; Yamamoto et al., 2002), we expect to observe a similar pathobiological role of iron in atypical presentations of AD. Therefore, the primary aim of this exploratory study was to investigate the regional relationship between magnetic susceptibility measured on QSM and tau uptake measured on PET in atypical AD to determine whether there is evidence that iron deposition may be related to the heterogeneity observed in tau deposition in AD. Considering the regional overlap between both modalities and the evidence of QSM-tau PET associations in the temporal lobe in amnestic AD (Spotorno et al., 2020), we hypothesized that we may observe local relationships, i.e., local regional colocalization between cortical tau uptake and increased susceptibility in key atypical AD regions such as temporo-parieto-occipital regions.

## 2 Methods

### 2.1 Patients

Forty patients with biomarker-confirmed AD who presented with visuospatial/perceptual (Crutch et al., 2017) (*n* = 24), language (*n* = 8) (Gorno-Tempini et al., 2011) or other atypical variants (dysexecutive AD = 3, motor = 4 and behavioral = 1) (Armstrong et al., 2013; Ossenkoppele et al., 2015; Townley et al., 2020) were recruited by the Neurodegenerative Research Group (NRG) from the Department of Neurology, Mayo Clinic, Rochester, MN, between September 30, 2020, and September 14, 2023. All patients were enrolled regardless of sex, race and ethnicity. They met clinical diagnostic criteria (as detailed above), underwent comprehensive neurological evaluations (KAJ or JGR), neuropsychological testing (MMM), and completed a structural MRI that included a five-echo gradient echo sequence for QSM calculation, [^11^C] Pittsburgh Compound-B (PiB) PET to confirm Aβ positivity and an [^18^F] AV-1451 PET scan to assess tau burden. All patients showed evidence of Aβ deposition on PiB-PET based on the established cut-offs for Aβ positivity (Jack et al., 2019). Patients were excluded if they were negative on either PET scan, had a stroke, tumor or structural brain abnormality that could explain their symptoms, or if they had poor vision (20/400).

### 2.2 Patient consent and protocols

This study was approved by the Mayo Clinic IRB. All patients gave written informed consent to participate.

### 2.3 Clinical testing

All patients underwent neurological and neuropsychological evaluations. The neurological tests included the Montreal Cognitive Assessment (MoCA) to assess general cognitive function (Nasreddine et al., 2005), Clinical Dementia Rating Scale – sum of boxes (CDR-SB) to assess global functional impairment (Hughes et al., 1982), Movement Disorders Society sponsored revision of the Unified Parkinson’s disease rating scale III (MDS-UPDRS III) to assess parkinsonism (Martinez-Martin et al., 1994), the Western Aphasia Battery ideomotor apraxia (WAB praxis) subtest to assess for ideomotor apraxia (Shewan and Kertesz, 1980), the Ishihara test to assess colour vision (Pache et al., 2003), the Cognitive Behavioral Inventory-revised version (CBI) subtest to evaluate presence or absence of hallucinations (Wear et al., 2008) and a battery of tests to assess simultanagnosia: (i) Ishihara color plates; (ii) images of overlapping line drawings, (iii) color images of complex picture scenes, and (iv) Navon figures, with performance scored on a 20-point scale (scores under 17 were considered abnormal based on performance in normal controls) (Brazis et al., 1998). The neuropsychological tests included the 15-item Boston Naming Test (BNT) to assess confrontation naming (Lansing et al., 1999), Boston Diagnostic Aphasia Exam (BDAE) repetition subtest to assess sentence repetition (Goodglass and Barresi, 2000), Visual Object and Space Perception Battery (VOSP) Cubes to assess visuospatial ability and VOSP Letters for assessing visuoperceptual ability (Warrington and James, 1991) and the Rey Auditory Verbal Learning Test—Recognition Percent Correct (AVLT-RPC) to measure episodic memory (Rey, 1958).

### 2.4 Image acquisition

Patients were scanned on 3 T volumetric MRI scanners (Magnetom Prisma, Siemens Healthineers) at Mayo Clinic, Rochester, MN. The scan included a 3D magnetization prepared rapid acquisition gradient echo (MPRAGE) sequence and a 3D multi-echo gradient echo sequence, as described previously (Singh et al., 2022; Satoh et al., 2024). For tau-PET, patients were injected with ~ 370 MBq (range 333–407 MBq) of [^18^F] AV-1451, followed by an 80 min uptake period. The acquisition was 20 min consisting of four, 5-min dynamic frames after a low-dose CT image. Detailed acquisition and preprocessing details have been previously published (Josephs et al., 2012; Whitwell et al., 2019; Satoh et al., 2024).

### 2.5 Image processing

All MRI and PET images were processed using NRG in-house developed pipelines (RS) as described previously (Satoh et al., 2024). First, unified segmentation (Ashburner and Friston, 2005) in SPM12 was used to determine the tissue probabilities of each MPRAGE scan with tissue priors and settings from the Mayo Clinic Adult Lifespan Template (MCALT) (Schwarz et al., 2017). Brain atlases were registered from the MCALT template space to native MPRAGE space using ANTs (Avants et al., 2008). The affine registration parameters were computed between the MPRAGE images and the first-echo magnitude GRE images. Laplacian-based phase unwrapping and background field removal were applied to the phase images (Wu et al., 2012). Improved sparse linear equations and the least squares method was then applied to compute the QSM from the processed phase images (Li et al., 2015). The QSM images were registered to the MPRAGE by using the affine parameters. The mean QSM signal was extracted from gray and white matter across the brain atlases. The tau-PET images were registered to their corresponding subject-space MPRAGE using SPM12. Mean tau-PET values were calculated for each region of interest (ROI) across gray and white matter. They were divided by the cerebellar crus gray matter median uptake value to generate standard uptake value ratios (SUVRs).

For the voxel-based analysis, images were spatially normalized into the template space using SPM 12 and then smoothed using a Gaussian kernel with 6 mm full width at half maximum. For the region-based analysis, the MCALT atlas (Schwarz et al., 2017) was used for the following ROIs: cortical ROIs included the superior frontal gyrus, superior temporal gyrus, middle temporal gyrus, inferior temporal gyrus, amygdala, hippocampus, entorhinal cortex, insula, posterior cingulum, precuneus, retrosplenial cortex, superior parietal gyrus, inferior parietal gyrus, angular gyrus, supramarginal gyrus, superior occipital gyrus, middle occipital gyrus and the inferior occipital gyrus. Subcortical ROIs included the caudate, putamen and pallidum. The Deep Brain Stimulation Intrinsic Template atlas (Ewert et al., 2018) was also used for the subthalamic nucleus, substantia nigra, and red nucleus, along with an in-house atlas (Whitwell et al., 2017) that was used for the cerebellar dentate.

### 2.6 Statistical analysis

The whole brain voxel-based regression analysis was performed using VoxelStats (Mathotaarachchi et al., 2016). A linear regression was fit in each voxel to evaluate the relationship between QSM and tau-PET while adjusting for age and sex. The analyzed regions were confined to the area where the averaged tissue mask for all patients exceeded 0.95. Results were presented using the *p* < 0.001 threshold and the Random Field Theory (RFT)-based multiple comparison correction with the cluster size threshold of *p* < 0.05 (Mathotaarachchi et al., 2016). For the ROI-based analysis, spearman’s partial correlation analysis was used for the cortical and subcortical ROIs while adjusting for age and sex. Results were corrected for multiple comparisons using False discovery rate (FDR). These plots were generated using MATLAB Statistics and Machine Learning Toolbox (R2022a). Scatterplots were generated to show the relationships between ROIs with the highest spearman rho. These plots were generated using Graph pad prism v10. Sensitivity analysis exploring the relationship between QSM and tau-PET within the visual variant was also performed.

### 2.7 Data availability statement

The data that supports the findings of this study will be available from the corresponding author on request via email.

## 3 Results

The clinical and demographic features of the cohort are shown in Table 1. The cohort had a median age of 64 years at the time of assessment, and 70% of them were female. Performance on the clinical tests was consistent with the patients’ AD diagnoses.

**Table 1:** Participant’s demographics and disease characteristics.

Demographics and disease characteristics	Atypical AD (*N* = 40)	Visual variant (*N* = 24)	Language variant (*N* = 8)	Other variants (*N* = 8)
Female, *n* (%)	28 (70%)	17 (70.8%)	5 (71.4%)	6 (66.7%)
Education, year	16 (12, 18)	16 (12, 18.5)	13 (12, 16)	14 (13.7, 16)
Age at scan, year	63.6 (61.6, 71.1)	62.7 (60.4, 67.4)	64.1 (62.6, 71.3)	64.4 (62.6, 71.7)
Age at onset, year	58 (55, 63)	58 (54, 60)	58 (57, 60)	64 (57, 69)
Disease duration, year	4.3 (3.1, 5.6)	4.3 (3.2, 5.3)	5.9 (4.8, 8.7)	3 (2.6, 3.2)
MoCA (30)	14 (9, 21)	17 (9.7, 22.2)	8 (6.2, 12.7)	16 (13.5, 18.7)
CDR-SB (18)	3.5 (1.5, 7.5)	4 (1.5, 6.7)	4 (1, 6)	2.5 (1.75, 5.75)
Simultanagnosia (20)	10 (5, 16.2)	6 (3, 9.2)	19 (17.5, 19)	16.5 (15.2, 17.7)
Ishihara (6)	1 (0, 4.2)	0 (0, 1)	5 (5, 5.75)	4.5 (3.2, 5.7)
VOSP cubes (10)	1 (0, 7)	0 (0, 1.5)	10 (6, 10)	4 (2, 9)
VOSP letter (20)	13 (6, 18.5)	6.5 (3, 14.5)	20 (19, 20)	19 (15, 20)
BNT-SF (15)	11 (6, 13)	12 (9.2, 14)	4.5 (2.75, 6.5)	11 (11, 13)
BDAE repetition (10)	9 (6, 10)	9 (7, 9.7)	3.5 (3, 4.5)	10 (9, 10)
CBI (180)	39 (15, 58.2)	50 (23.7, 62)	26 (13, 59)	25 (14, 38)
MDS-UPDRS III (132)	4 (0.25, 6)	4 (0.25, 6)	5 (3, 7.5)	5 (2, 12)
WAB praxis (60)	58 (55, 60)	59 (55, 60)	57 (50.7, 59.5)	55 (55, 55)
AVLT RPC (100)	73.4 (66.7, 86.7)	78.4 (68.4, 90)	70 (61.7, 78.4)	66.7 (61.7, 76.7)
Tau-PET SUVR composite	2.13 (1.76, 2.36) ± 0.44	2.25 (2.01, 2.46) ± 0.49	2.11 (1.71, 2.35) ± 0.56	2.02 (1.84, 2.17) ± 0.46
QSM susceptibility composite	0.002 (0.001, 0.002) ± 0.0009	0.002 (0.0009, 0.002) ± 0.001	0.002 (0.001, 0.002) ± 0.001	0.002 (0.001, 0.003) ± 0.001

Data shown are *n* (%) or median (first and third quartiles). Standard deviations have been included for imaging metrics. The imaging composites were created by calculating a weighted average of the temporal (superior, middle and inferior gyri), occipital (superior, middle and inferior gyri) and parietal (superior, inferior, angular and supramarginal gyri) regions. AVLT RPC, Auditory Verbal Learning Test – Recognition Percent Correct; MoCA, Montreal Cognitive Assessment Battery; CBI, Cambridge Behavioral Inventory; CDR-SB, Clinical Dementia Rating Scale- sum of boxes; BNT-SF, Boston Naming Test Short Form; BDAE, Boston Diagnostic Aphasia Exam; MDS-UPDRS III, Movement Disorders Society sponsored revision of the Unified Parkinson’s disease rating scale III; VOSP, Visual Object and Space Perception Battery; WAB, Western Aphasia Battery.

**Figure 1:**
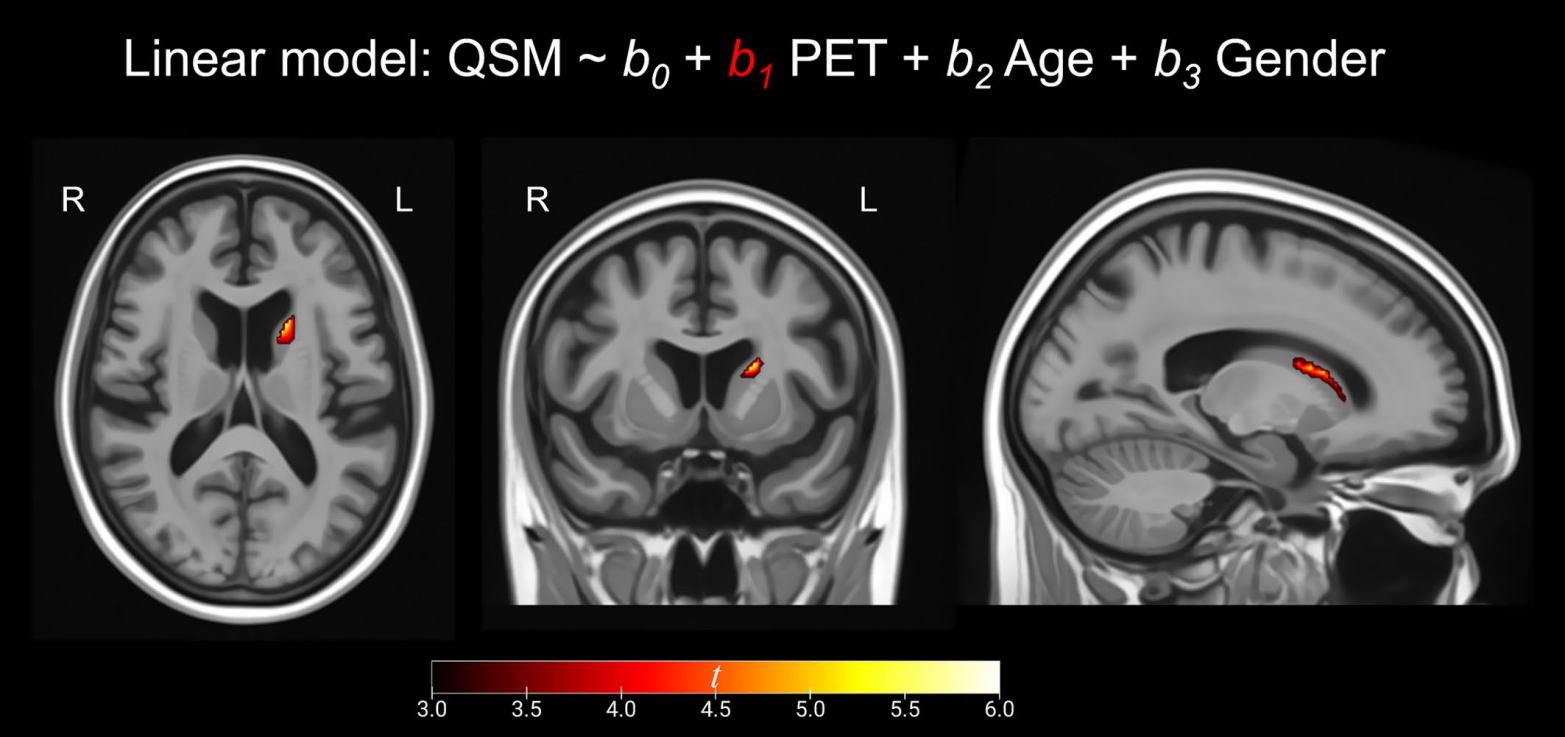
Voxel-based analysis. These maps represent the positive correlation between susceptibility and tau PET SUVR as calculated by linear regression calculated for each voxel. Results are presented at *p* < 0.001 using the random field theory (RFT)-based multiple comparison correction. MRIcroGL was used for visualization. The scale represents t scores ranging from +3 to +6.

**Figure 2:**
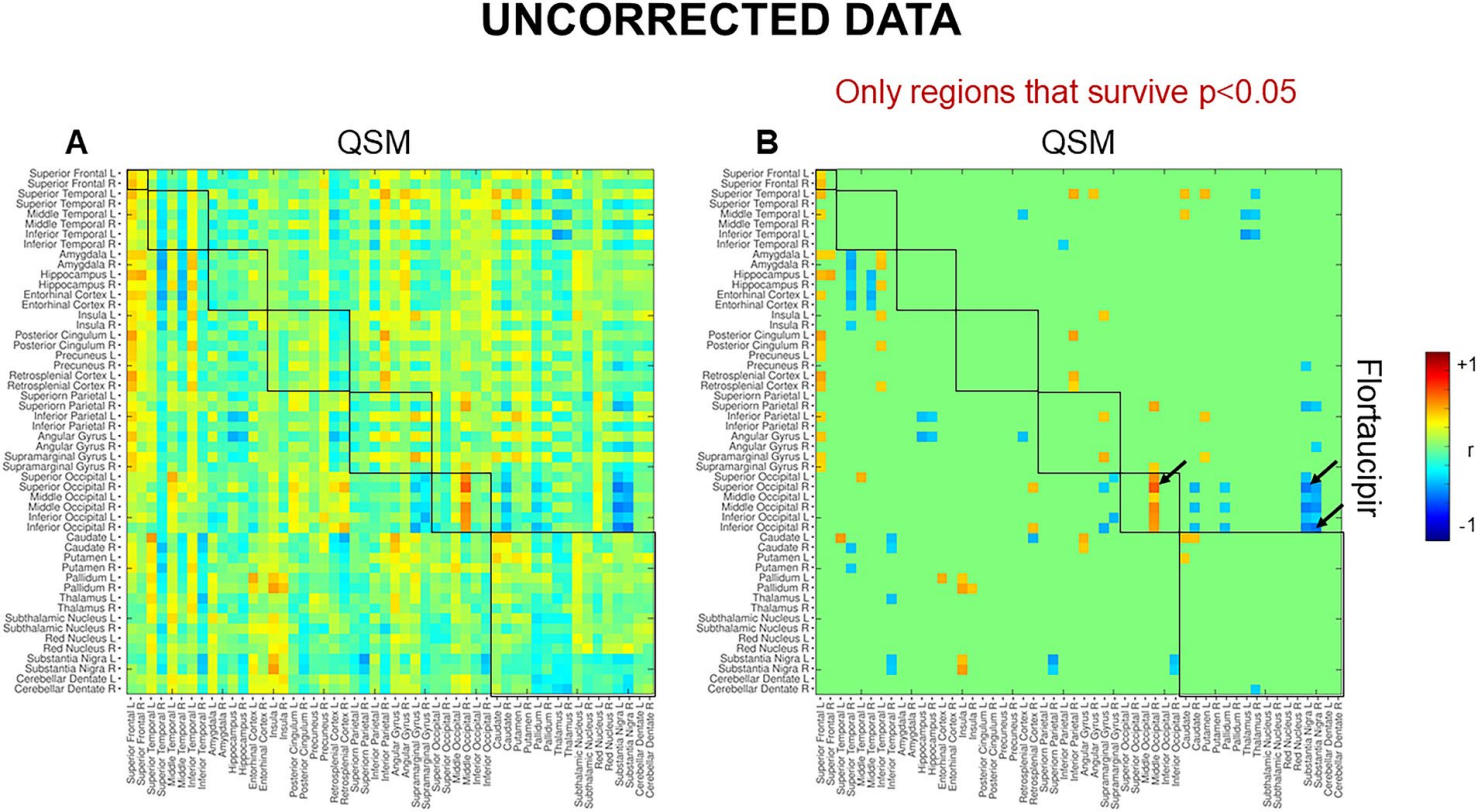
Region-based analysis. These maps represent the results for spearman correlation. **(A)** Uncorrected spearman correlations and **(B)** uncorrected spearman correlations showing only regions that survive *p* < 0.05. The scale represents spearman rho values ranging from −1 to +1. No significant correlations were noted after correcting for multiple comparisons using FDR.

**Figure 3:**
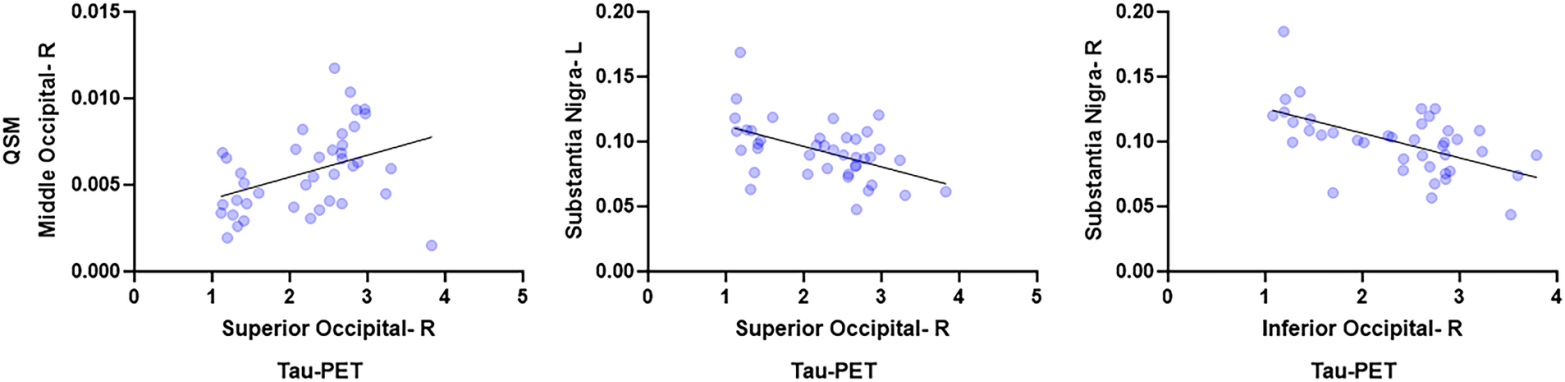
Region-based analysis. These scatterplots show the relationships between ROIs with the highest spearman rho. These plots were generated using Graph pad prism v10.

At the voxel-level, positive correlations were observed between magnetic susceptibility and tau-PET uptake in the left caudate after the RFT-based multiple comparison correction (Figure 1). At the ROI-level, no significant correlations were found after correction for multiple comparisons. However, uncorrected data showed a moderate association (absolute Spearman rho ≥0.50) in three ROI-pairs (shown with arrows) (Figure 2), namely between tau-PET uptake in the right superior occipital lobe and magnetic susceptibility in the right middle occipital lobe (r = 0.57, *p* < 0.001, FDR-corrected *p* = 0.59) and the left substantia nigra (r = −0.50, *p* < 0.01, FDR-corrected *p* = 0.96), and between tau-PET uptake in the right inferior occipital lobe and magnetic susceptibility in the right substantia nigra (r = −0.53, p < 0.001, FDR-corrected *p* = 0.95) (Figures 2, 3). Spearman rho for the relationship between susceptibility and tau-PET uptake in the left caudate was 0.37, *p* = 0.02, FDR-corrected *p* = 0.96. The sensitivity analyses within the visual variant showed no significant voxel-level clusters and ROI-level correlations after correction for multiple comparisons. The uncorrected ROI-level data showed an association between tau-PET uptake in the left superior occipital lobe and magnetic susceptibility in the left middle (r = 0.53, *p* = 0.01, FDR-corrected *p* > 0.99) and superior temporal lobes (r = 0.52, *p* = 0.01, FDR-corrected *p* > 0.99) (Supplementary Figure 1).

## 4 Discussion

This study examined the relationship between iron burden measured on QSM and tau accumulation measured on AV-1451 PET in atypical clinical presentations of AD. We found some weak evidence for positive local relationships between susceptibility and tau-PET in the caudate nucleus and occipital lobe.

The whole brain voxel-level analysis revealed a positive correlation between magnetic susceptibility and tau-PET uptake in the left caudate. This relationship was also significant, albeit weak, at the ROI-level. Greater magnetic susceptibility in the caudate has been noted in the language variant of AD when compared to cognitively unimpaired individuals (Singh et al., 2022). However, the caudate does not typically show elevated tau-PET uptake in the visual, language, behavioral, and dysexecutive variants of AD (Ossenkoppele et al., 2016; Tetzloff et al., 2018), although it can be affected in the motor variant of AD (Smith et al., 2017). It is important to note that of the 40 atypical AD patients in this study, only three patients were diagnosed with the motor variant and eight were diagnosed with the language variant of AD. One explanation for this finding could be that basal ganglia structures, including the caudate, are known to be “off-target” sites for AV-1451 tracer uptake (Marquie et al., 2015; Johnson et al., 2016), with off-target binding increasing with older age. Moreover, iron levels are known to increase steadily with age, particularly after middle age in the caudate nucleus (Hallgren and Sourander, 1958; Ramos et al., 2014). Likewise, previous studies have also shown that AV-1451 PET and iron-sensitive MR (R2* or QSM) signals positively correlated in the caudate, putamen, and pallidum in healthy controls, which is an unexpected finding as these individuals should not have iron-related tau deposition (Choi et al., 2018; Satoh et al., 2024). In our previous study, we performed the same voxel-based analysis on 67 cognitively normal controls and found positive correlations between tau PET and QSM in the bilateral caudate and pallidum (Satoh et al., 2024). Therefore, the presence of positively correlated signal between the two modalities in the caudate may be reflective of off-target binding to iron rather than biological relationships, such as the coexistence or the interaction between iron and tau (Yamamoto et al., 2002; Spotorno et al., 2020). The reason for a leftward asymmetry is unclear but has been reported before in the caudate nucleus (Holz et al., 2022).

No significant associations were noted between magnetic susceptibility and tau-PET uptake at the ROI-level after correction for multiple comparisons. However, uncorrected data showed a positive correlation between modalities in the occipital lobe. Although this finding is consistent with the spatial distribution of tau accumulation (Day et al., 2017; Singh et al., 2024) and greater occipital susceptibility reported in the visual and language variants of AD (Singh et al., 2022), the correlation is weak and does not survive correction. One could theorize the presence of a mechanistic relationship between iron and tau deposition in the occipital lobe in atypical AD, but these findings will have to be confirmed in a larger cohort. Lastly, the sensitivity analyses showed no significant associations after correction for multiple comparisons, but the uncorrected data showed an association between the occipital tau uptake and temporal susceptibility in the visual variant of AD. One could theorize that this finding could be suggesting toward a relationship between iron and tau deposition, as previous literature has shown significant tau uptake in occipital lobe (Day et al., 2017) and greater temporal lobe susceptibility (Singh et al., 2022) in visual AD. However, this subgroup analysis was underpowered, and the uncorrected results may include false positives, which should be interpreted cautiously. Overall, no positive evidence was found even in the best-case exploratory scenario once corrected, supporting the overall null finding.

A somewhat counterintuitive negative relationship was observed between substantia nigra susceptibility and occipital tau-PET uptake, whereby lower substantia nigra iron burden was associated with greater tau uptake in the occipital lobe. Substantia nigra is a major site for iron storage and is known to show a steady increase in iron levels with aging (Zecca et al., 2001). We have observed increased susceptibility in the substantia nigra in the language variant of AD but not the visual variant of AD (Singh et al., 2022). The reason for a negative association with occipital tau-PET uptake is unclear. This finding, although uncorrected, could be suggesting that iron deposition in brainstem nuclei may have a different mechanistic relationship with tau compared to iron in the cortex in AD. It is possible that susceptibility findings in cortical regions are influenced by myelin loss and white matter damage, whereas this is not expected to be a confound in brainstem grey matter nuclei. However, since the correlation did not survive correction for FDR, we suggest interpreting this finding cautiously and replicating these results in a larger cohort.

Strengths of this study include the consistent neuroimaging protocols and clinical evaluations with models that control for differences in age and sex effects. This is also the first study that directly compares the relationship between iron content measured on QSM and tau accumulation measured on AV-1451 PET in atypical clinical presentations of AD. Potential limitations include the relatively small sample size and the lack of power to examine relationships within each AD variant separately. Another limitation is the presence of off-target AV-1451 PET uptake (Marquie et al., 2015; Johnson et al., 2016) and age-related iron accumulation (Hallgren and Sourander, 1958; Ramos et al., 2014) in the basal ganglia structures. More specifically, the AV-1451 PET ligand is known to bind with iron-rich area in the basal ganglia, particularly to neuromelanin, which is known to accumulate with age (Marquie et al., 2015; Lowe et al., 2016; Choi et al., 2018; Langley et al., 2024). Additionally, AV-1451 PET and QSM signals are known to be positively correlated in the basal ganglia of healthy controls, suggesting toward iron-related tau deposition in these individuals (Choi et al., 2018; Satoh et al., 2024). Together, these issues make interpretation of the caudate findings challenging and raise questions on the existence of meaningful QSM-tau relationships and their utility in the deep nuclei. Lastly, our cohort consist predominantly of the language and visual variants of AD, so further studies will be needed to determine whether these findings can be generalized to cohorts with different phenotypic distributions. The reproducibility of these findings will also have to be confirmed in a larger cohort. Multi-site data aggregation would be needed to draw firm and comprehensive conclusions about this relatively rare disorder with diverse phenotypes.

Overall, these results show little evidence to suggest that iron deposition is strongly related to or driving regional patterns of tau deposition in atypical clinical presentations of AD. More work is needed in larger cohorts to better understand the pathophysiological role of iron in AD.

## Data Availability

The raw data supporting the conclusions of this article will be made available by the authors, without undue reservation.

## Supplementary material

The Supplementary material for this article can befound online at: https://www.frontiersin.org/articles/10.3389/fnagi.2025.1615718/full#supplementary-material

SUPPLEMENTARY FIGURE 1 Region-based analysis. These maps represent the results for spearman correlation for the visual variant. The panel shows uncorrected spearman correlations with only regions that survive *p* < 0.05.

## Glossary

AD: Alzheimer’s disease
Aβ: beta amyloid
MCALT: Mayo Clinic Adult Lifespan Template
MRI: Magnetic resonance imaging
MPRAGE: Magnetization prepared rapid gradient echo
NRG: Neurodegenerative research group
PiB: Pittsburgh compound B
PET: Positron emission tomography
ROI: Regions of interest
QSM: Quantitative susceptibility mapping
SUVRs: Standard uptake value ratios
MoCA: Montreal cognitive assessment
CDR-SB: Clinical dementia rating scale—sum of boxes
MDS-UPDRS III: Movement disorders society sponsored revision of the unified Parkinson’s disease rating scale III
WAB: Western Aphasia battery
CBI: Cognitive behavioral inventory-revised version
BNT: Boston naming test
BDAE: Boston diagnostic aphasia exam
VOSP: Visual object and space perception battery
AVLT-RPC: Rey auditory verbal learning test-recognition percent correct
