# Supplementary figures and images for: Relationship between tau-PET and quantitative susceptibility mapping in atypical Alzheimer’s disease
Source: Front Aging Neurosci. 2025 Jul 3;17:1615718. doi: 10.3389/fnagi.2025.1615718 (PMC12267165; doi:10.3389/fnagi.2025.1615718)

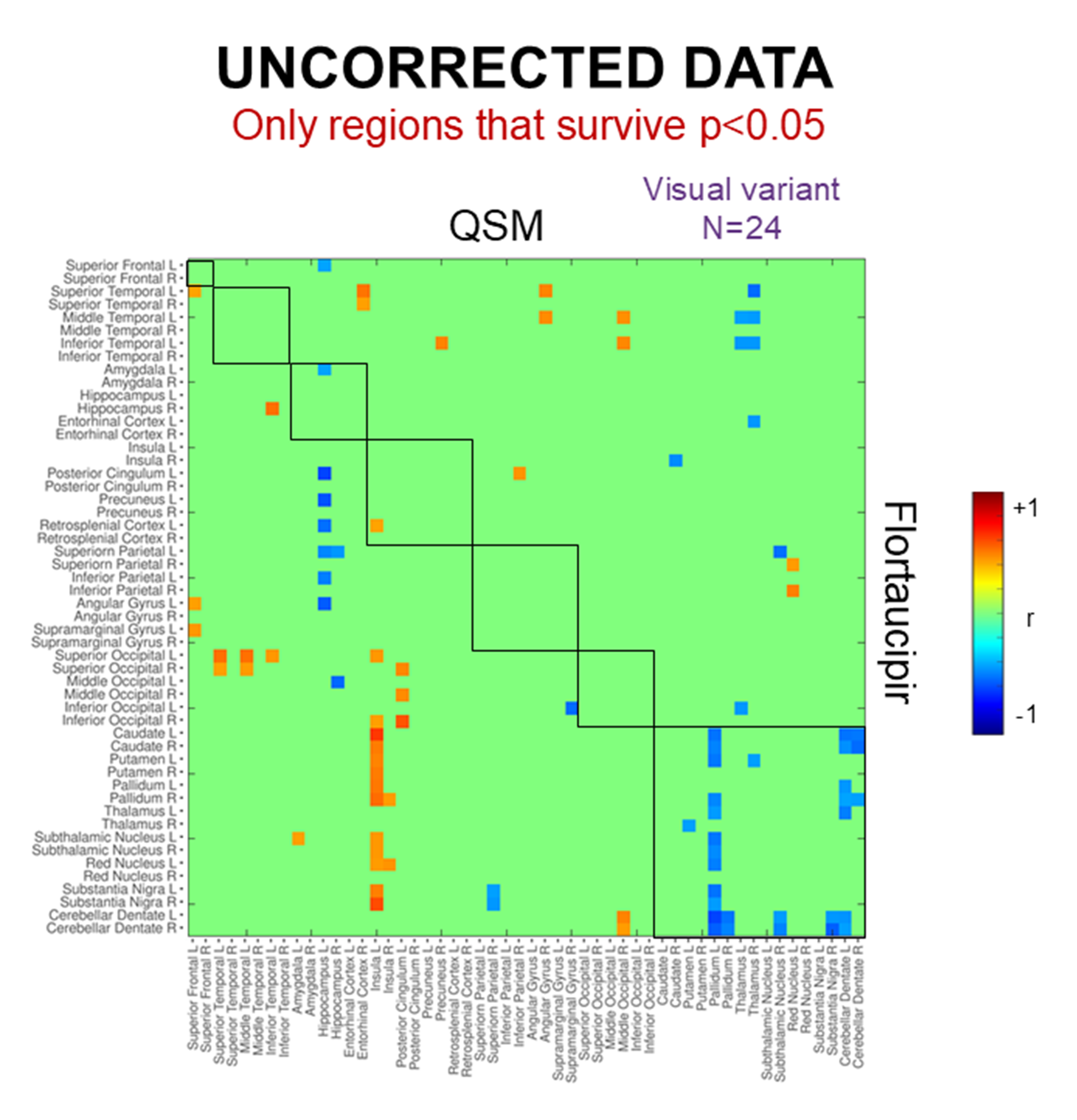

Supplement: SUPPLEMENTARY FIGURE 1 — Region-based analysis. These maps represent the results for spearman correlation for the visual variant. The panel shows uncorrected spearman correlations with only regions that survive p < 0.05. [file Image_1.tif]
